# Variation in fiberoptic bead-based oligonucleotide microarrays: dispersion characteristics among hybridization and biological replicate samples

**DOI:** 10.1186/1745-6150-1-18

**Published:** 2006-06-20

**Authors:** Jaroslav P Novak, Merrill C Miller, Douglas A Bell

**Affiliations:** 1 McGill University and Genome Quebec Innovation Centre, 740 Dr. Penfield, Montreal, Quebec, H3A 1A4, Canada; 2 Environmental Genomics Section, Laboratory of Molecular Genetics, C3-03, PO Box 12233, National Institute of Environmental Health Sciences, Research Triangle Park, NC 27709, USA

## Abstract

**Background:**

Gene expression microarray technology continues to evolve and its use has expanded into all areas of biology. However, the high dimensionality of the data makes analysis a difficult challenge. Evaluating measurements and estimating the significance of the observed differences among samples remain important issues that must be addressed for each technology platform. In this work we use a consecutive sampling method to characterize the dispersion patterns of data generated from Illumina fiberoptic bead-based oligonucleotide arrays.

**Results:**

To describe general properties of the dispersion we used a linear function SD = a + bY_mean_, approximating the standard deviation across arrays (Y_mean _is the mean expression of a given consecutive sample). First we examined three levels of variability: 1) same cell culture, same reverse transcription, duplicate hybridizations; 2) same cell culture, reverse transcription replicates; 3) parallel cultures. Each higher level is expected to introduce a new source of variability. We observed minor differences in the constant term: the mean values are 3.5, 3.1 and 3.5, respectively. However, the mean coefficient b increased from 0.045 to 0.147 and 0.133. We compared the coefficients derived from the consecutive sampling to those obtained from the standard deviation of individual gene expressions and found them in good agreement. In the second experiment samples we detected 11 genes with systematically different expressions between the experiment samples treated with glucose oxidase and controls and corroborated the selection using the Mann-Whitney and other tests. We also compared the consecutive sampling and coincidence method to t-test: the average percentage of consistency was above 80 for the former and below 50 for the latter.

**Conclusion:**

Our results indicate that the consecutive sampling method and standard deviation function provide a convenient description of the overall dispersion of Illumina arrays. We observed that the constant term of the standard deviation function is at average approximately the same for duplicate hybridization as for the assays with additional sources of variability. Furthermore, among the genes affected by glucose oxidase treatment we identified 6 genes in oxidative stress pathways and 5 genes involved in DNA repair. Finally, we noted that the consecutive sampling and coincidence test provide, under given conditions, more consistent results than the t-test.

**Reviewers:**

This article was reviewed by Alexander Karpikov (nominated by MarkGerstein), Jordan King and Eugene V. Koonin.

## Open peer review

Reviewed by Alexander Karpikov (nominated by Mark Gerstein), Eugene V. Koonin, and Jordan King. For the full reviews, please go to the Reviewers' comments section.

## Background

The usefulness of DNA microarray technology in the exploration of gene expression profiles can hardly be overstated. Along with the dramatic increase in microarray publications (a 2.5-fold increase per year since 1997, to >3,000 in 2004) and a broadening in the scope of applications, the methods of analysis of microarray data have grown in variety and sophistication, from simple fold-difference criteria to complex Bayesian procedures and clustering techniques [1-9]. In spite of these advances, evaluating variation and estimating the significance of the observed differences in recorded signals remain a difficult challenge. Existing methods provide various approximations of reality, balancing Type I against Type II error, but none can be considered ideal under all conditions. This is mainly due to the inherent complexity of the problem, but is sometimes due to the use of oversimplified conditions. Mehta et al. [10] offered an interesting overview of the subject, including the discussion of misconceptions about generality and applicability of some approaches.

Quantities that are taken as a measure of gene expression are affected by number of processes that contribute to variation, resulting in the random and/or pseudorandom component of the signal. Such variation may be separable into the "technical component," caused by the technical factors, such as variability of experimental protocols, autofluorescence and backscatter, laser-molecule interactions, photomultiplier noise etc., and the "sampling" or "physiological component," which depends mainly on the variability caused by the differences between the samples, e.g. differences in the biological state or purity of the sample composition. Distinguishing if a given gene expression intensity value is greater than the background noise or is different between two samples are fundamental issues in microarray analysis.

For the single-color Affymetrix arrays we have two groups of methods aiming at separation of the true signal from the random components: "low level" and "high level." The former approach deals with the fluorescence signals of each individual probe and includes background correction, adjustment for the nonspecific signal and expression summary that yields an approximation of RNA abundance or "gene expression," the latter takes the gene expression as an elementary variable [11]. Low-level analysis can be used only when a relatively large number (say 8 or more) of probes or probe pairs per probe set is available. Moreover, the standard methods, such as dChip [12,13] or RMA [14-16], are not applicable if only duplicates are available and not quite reliable for triplicates (URL address for the RMAExpress is ).

The high level analysis consists of two basic steps: normalization and statistical evaluation of the observed differences. One approach to normalization relies on the "reference genes" (e.g. [17-20]), but genes providing "ubiquitous reference" are hard to find [21] and they require an additional experimental effort. The other calculates normalization coefficients from the expression values. In case of linear dependence between the measured signal and RNA abundance and balanced over- and under-expressed values, the global normalization is suitable. In case of nonlinearity, LOWESS [22] or other appropriate correction has to be employed [13]. Statistical significance of the observations is often estimated using standard parametric tests, such as the t-test or ANOVA. However, a certain percentage of the frequency distributions always deviates from the normal distribution and in multiple comparisons of thousands of gene expressions this can lead to a substantial error. Furthermore, number of replicates is usually small and estimated variances often differ largely from the true value. Novak et al. suggested characterization of dispersion patterns of Affymetrix arrays with the method of consecutive sampling [23], which uses groups of genes with close mean expressions to estimate the standard deviations; similar approach was independently proposed by Baldi and Long [24] and Kamb and Ramaswami [25]. Two component model including the constant and proportional terms of the standard deviation was introduced by Rocke and Lorenzato [26] in the context of analytical chemistry and later applied to cDNA and oligonucleotide microarrays [27]; see also [28,29]. Choe et al. [11] compared performance of the t-test, modified t-test developed by Tusher et al. [30] and method of Baldi and Long [24] and concluded that the last method showed, under given conditions, superior performance. Some other approaches were also suggested and tested. For example, Troyanskaya et al. [31]examined three nonparametric methods, Durbin et al. [32] proposed a variance-stabilizing transformation and Bilke et al. [33] used Bayesian approach. Among other publications, the paper by McClinick et al. [34], e.g., deals with reproducibility of microarray data, Kooperberg et al. [35] compared several statistical methods and Jarvinen et al. [36] different microarray platforms.

Many new microarray-based platforms are available and some, which allow parallel analysis of many samples, may be suitable for high throughput analysis. Here we utilized the Illumina GEX Sentrix™ Array Matrix (SAM) system to evaluate gene expression for 632 genes in 96-well format. Our first aim is to characterize expression data and assess various sources of dispersion. We describe the data obtained from replicate hybridizations, reverse transcription reactions, and biological cultures and evaluate the frequency distributions. Subsequently, we compare the dispersion patterns, and assess the contribution of each additional process to variability of data. The second aim is to study systematic differences in gene expression values in the control cell cultures and cell cultures subjected to a particular treatment. We analyze the data from a cell line subjected to a continuous low-dose oxidative stress exposure (~10 μm H_2_O_2_) for 24 hrs. In our analysis we use the consecutive sampling method [23], which quantifies dispersion between two samples by ranking the probe sets according to the mean signal intensity, grouping them in bins containing *k *consecutive gene pairs, and calculating standard deviations from the difference of expressions (in this study *k *= 12). We search for the best candidate genes affected by the treatment among the differentially expressed genes, using the consecutive sampling and coincidence test. The results are compared to the t-test and Wilcoxon (Mann and Whitney) nonparametric test. In addition, we examine consistency of the results obtained by the coincidence test and compare to the t-test on normalized data, log-transformed data and data subjected to the variance stabilization transformation, to the method of analysis by Tusher et al. [30] and to Baldi and Long [24] CyberT method.

## Results

### Experimental approach

Illumina GEX Sentrix™ Array Matrix utilizes oligonucleotides attached to 3 micron beads immobilized on fiber optic bundles. Each oligonucleotide probe is represented on ~30 beads per array, allowing averaging of many signals for the same oligonucleotide probe and a consequent reduction in signal variation. Each gene typically has two probes and the intensity signal for a given gene (or probe set), is the average of the available probe signals. The consecutive sampling approach was used to characterize the dispersion patterns of the gene expression data obtained from oligonucleotide probes for 632 genes on three groups of samples: 1) RNA samples from five parallel cultures of lymphoblast cell line GM10469 were reverse transcribed and each has a hybridization duplicate (Samples C1–C5); 2) Pooled reference RNA sample has three reverse transcription/cRNA replicates (PR1, PR2, PR3, each with a duplicate hybridization); 3) Parallel cultures of lymphoblast cell line GM12831 were grown either untreated or in the presence of 1 mUnit/ml glucose oxidase (which generated a continuous dose of ~10 μmole H_2_O_2_). The RNA from each of these 4 samples (GN1, GN2, GO1, GO2) was reverse transcribed and hybridized to arrays in duplicate. The data from these hybridizations allows evaluation of variation due to hybridization, reverse transcription, and parallel biological cultures. We used a coincidence test to identify differentially expressed genes in treated samples and compared these results with several other approaches.

### Normalization and frequency distributions

Prior to the data analysis we calculated the gene expression per probe set by averaging the signals of the available probes. Subsequently, all the datasets were normalized to 100% of the total mean expression across the array. Dispersion plots and running mean plots of the pair-wise comparisons showed, in most cases, deviations from the 45° line at the low expression end; the additive correction constants range from about -5 to +2 (normalized values). Additional files 1 to 4 illustrate the effect of correction on a particular example of the dispersion plots for paired hybridization replicates C5a and C5b (Additional file 1: Dispersion patters before normalization, Additional file 2: Running mean before normalization, Additional file 3: Dispersion pattern after normalization, Additional file 4: Running mean after normalization). In some cases we noted a saturation effect, which was corrected by power functions.

### Characterization of variability: frequency distributions and standard deviation functions

**Figure 1:**
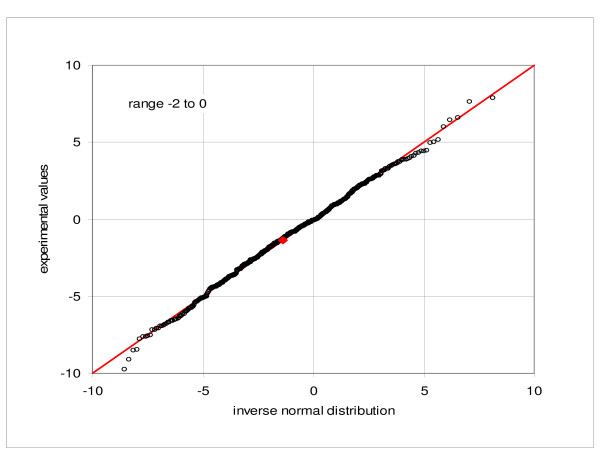
**Quantile-quantile plot of the frequency distribution**. Comparison of the observed expressions with the corresponding inverse normal distribution, combined samples C1a, C2a, C3a, C4a, C5a: range of average expressions from -2 to 0.

**Figure 2:**
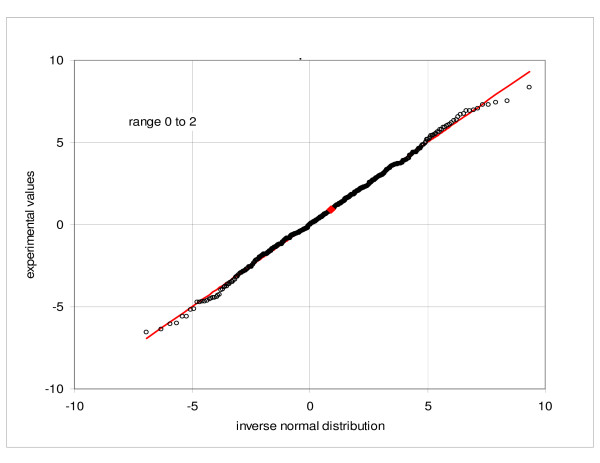
**Quantile-quantile plot of the frequency distribution**. Comparison of the observed expressions with the corresponding inverse normal distribution, combined samples C1a, C2a, C3a, C4a, C5a: range of average expressions from 0 to 2.

**Figure 3:**
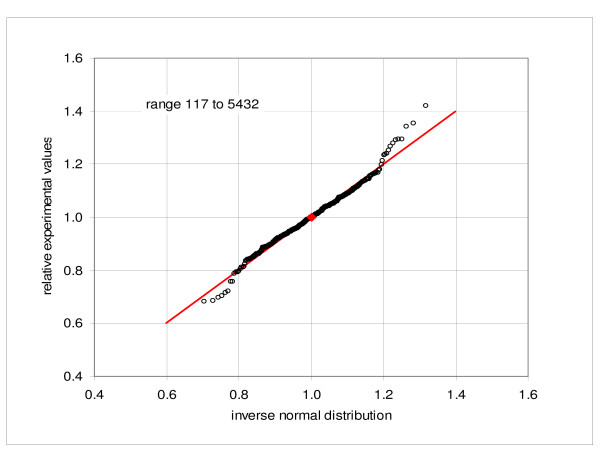
**Quantile-quantile plot of the frequency distribution**. Comparison of the observed expressions with the corresponding inverse normal distribution, combined samples C1a, C2a, C3a, C4a, C5a: range of average expressions from 117 to 5432; figure shows the relative values (expressions divided by the mean of five arrays).

We examined properties of our data by sampling the combined expression values for the first replicate hybridization comprising five parallel cultures of the cell line GM10469 (C1a, C2a, C3a, C4a, C5a) and comparing them to the normal frequency distribution. Figures 1 and 2 show quantile-quantile (Q-Q) plots of the observed values versus the corresponding inverse normal distribution at the low-end of mean intensities, from -2 to 0 and from 0 to 2, respectively. As expected, the distribution has the same character at the positive and negative side of zero. Only about three outlying points are noted in each figure. Figure 3 then shows Q-Q plot of the relative expressions (measured expressions divided by the mean of five samples) in the range of mean expressions from 117 to the maximum of 5432. Here about 396 out of 415 points lie very close to the normal reference, while the remaining 19, corresponding to about 4.5%, deviate from the diagonal. Similar results are obtained when using the set C1b, C2b, C3b, C4b and C5b. Verification of the normality is a quality check, incorporated into the consecutive sampling program (see Methods). For example, in the consecutive sampling of duplicate hybridizations and biological culture replicates, an average 6.8% and 5.0% of the samples failed the Kolmogorov-Smirnov test at the level of 0.05.

Conformity to the normal distribution is an important property. For a normally distributed population the quality of a given population sample can be assessed by comparing the sample frequency distribution to the normal distribution function. Analysis of frequency distribution of the observations also provides information about the character of random processes. In our particular case we note that in the low range the distribution function of expression values agrees well with the normal distribution, while in the high range the distribution of relative expressions is close to the normal. On the other hand, distribution of the relative expressions in the low-expression region and of the observed values in the high range deviate substantially from the normal form (see Additional files 5 and 6: Quantile-quantile plot of the frequency distribution). This corroborates the proposition that the standard deviation of the random variability consists of two components: a constant term and a directly proportional term, as suggested (e.g. Novak et al. [23]). It follows further that the standard deviation can be well represented by a linear characteristic function with a constant term. It is also important to note, that the negative observations are meaningful and the observations are symmetrically distributed around zero; the standard deviation of the statistical samples in near-zero region provides the best approximation of the constant term of the standard deviation function. Finally, the normal distribution is a necessary condition for application of the parametric methods, although the normality assumption is rarely, if ever, verified (Pavelka et al. [37] is a particular exception).

### Variation between duplicate hybridizations

We examined the dispersion in Illumina array data obtained from duplicate hybridizations of RNA samples extracted from 5 parallel cultures of the cell line GM10469 (pairs: C1a vs. C1b, C2a vs. C2b, C3a vs. C3b, C4a vs. C4b, C5a vs. C5b). For each pair we determined the standard deviation of the consecutive samples and fitted the calculated values to the characteristic function

**Figure 4:**
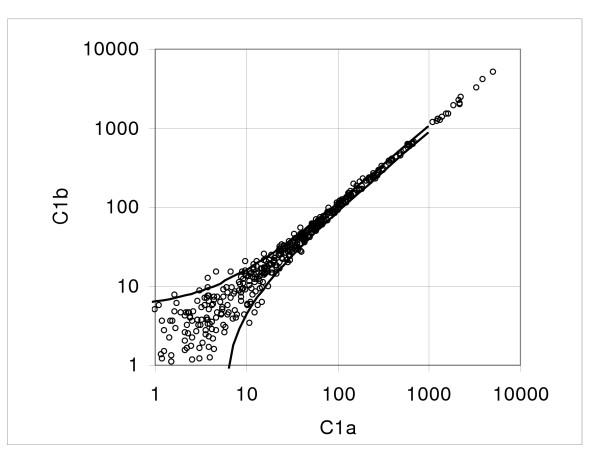
**Example of dispersion in the duplicate hybridization assay**. Dispersion plot of the pair C1a versus C1b. Figure shows the experimental points and boundaries of the 0.9 probability interval.

**Figure 5:**
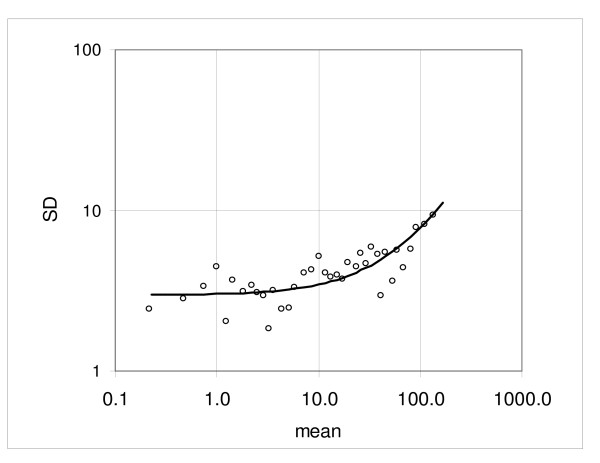
**Example of dispersion in the duplicate hybridization assay**. Standard deviation calculated from the consecutive samples and the regression curve representing the standard deviation function.

*SD *= *a *+ *b Y*_*mean*_,     (1)

at the logarithmic scale; here *Y*_*m *_is the sample mean (see the Method section, Method of analysis). The coefficient values calculated for the individual pairs and the mean of five pairs are shown in Table 1 (Hybridization 1 vs. hybridization 2) and a typical low dispersion pair C1a vs. C1b is plotted in Figure 4; Figure 5 shows the experimental standard deviations and the regression curve, representing the characteristic standard deviation function (1). For calculation of the characteristic function we exclude the top 10 samples to keep the variability of mean expression within the samples small. Furthermore, at the low end the expression range is limited by requirement that the mean value must be positive.

**Table 1:** Table of the mean coefficients of standard deviation characteristic function. The table shows the average values for parallel hybridization, parallel biological cultures and pooled reference sample. The coefficients *a *and *b *are the coefficients of the standard deviation function; CV stands for the coefficient of variation.

Comparison	*a*	*b*
GM10469 Hybridization 1 vs hybridization 2		

Hybridization 1 vs. 2: C1a vs C1b	2.97	0.048
Hybridization 1 vs. 2: C2a vs C2b	3.24	0.040
Hybridization 1 vs. 2: C3a vs C3b	4.27	0.034
Hybridization 1 vs. 2: C4a vs C4b	3.71	0.037
Hybridization 1 vs. 2: C5a vs C5b	3.54	0.074
Average (n = 5 pairs)	3.54	0.047
Coefficient of variation	0.14	0.35
Combined Series (C1a – C4a) vs (C1b-C4b)	3.51	0.043

Biological replicates		

Series Ca, all pairwise hyb 1; C1a-C5a		
Average (n = 10)	3.86	0.130
Coefficient of variation	0.08	0.27
Series Ca, mean based on individual genes (n = 5)	3.39	0.120
Series Cb, all pairwise hyb 2; C1b-C5b		
Average (n = 10)	3.08	0.136
Coefficient of variation	0.05	0.31
Series Cb, mean based on individual genes (n = 5)	3.00	0.116

Hybridization variation		

Pooled reference RNA (PR1a vs PR1b)	2.12	0.045
Pooled reference RNA (PR2a vs PR2b)	3.52	0.082
Pooled reference RNA (PR3a vs PR3b)	4.86	-0.003
Pooled reference RNA, hybridization 1 vs 2		
Average (n = 3 pairs)	3.50	0.042
Coefficient of variation	0.39	0.61

Transcription and hybridization variation		

Series 1		
PR1a vs PR2a	2.90	0.128
PR1a vs PR3a	3.30	0.130
PR2a vs PR3a	4.45	0.103
Average (n = 3 pairs)	3.55	0.120
Coefficient of variation	0.23	0.13

Series 2		

PR1b vs PR2b	3.20	0.184
PR1b vs PR3b	3.33	0.118
PR2b vs PR3b	4.68	0.153
Average (n = 3 pairs)	3.73	0.152
Coefficient of variation	0.22	0.22

To estimate the effect of the samples size we also evaluated the standard deviation function using *k *= 24. Since on given Illumina arrays the number of probe sets is relatively small, we evaluated the coefficients of standard deviation function in comparisons of the combined samples C1a to C4a versus C1b to C4b; for *k *= 24 we obtained *a *= 3.66 and *b *= 0.044, about 4.4% and 3.4% above the values *a *= 3.51 and *b *= 0.043 obtained for *k *= 12 and shown in Table 1. Since the spread of mean expression values is larger in larger samples, we expect higher dispersion. Furthermore, the intercept and the coefficient of proportionality obtained from the combined series (*k *= 12) are just about 1 and 8 percent below the means of the individual pair-wise comparisons 3.54 and 0.047, respectively (Table 1). Again, given that variance of the consecutive samples is enhanced by spread of the mean expression values, we expected smaller coefficients of the standard deviation function in combined comparison, which has higher density of the mean expression values. The coefficients of variation for *a *and *b *are 0.14 and 0.35, respectively. Of note, in this group the values of the intercept *a *are quite similar across the five-pair set; however, the coefficient of proportionality *b *for the pair C5a versus C5b is 0.074, about double of the mean of remaining pairs (0.040).

**Figure 6:**
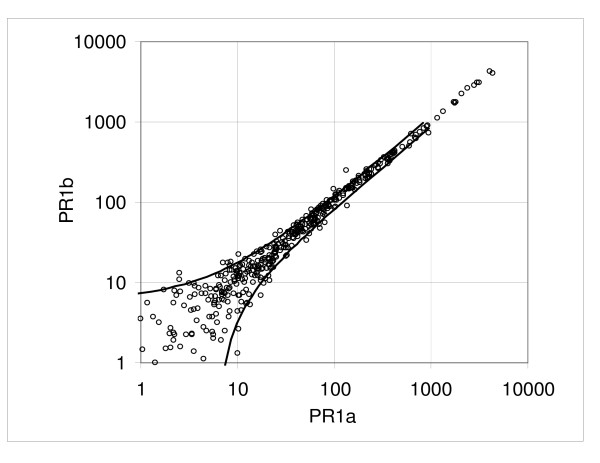
**Example of dispersion in the pooled RNA reference assay: pair PR1a versus PR1b**. Experimental points and boundaries of the 0.9 probability interval.

**Figure 7:**
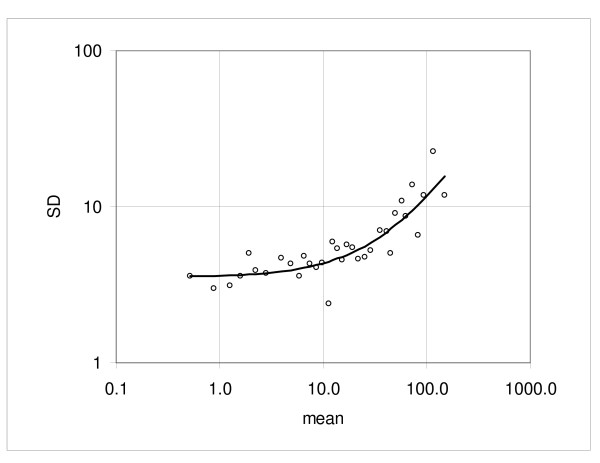
**Example of dispersion in the pooled RNA reference assay: pair PR1a versus PR1b**. Standard deviation calculated from the consecutive samples and the regression curve representing the standard deviation function.

**Figure 8:**
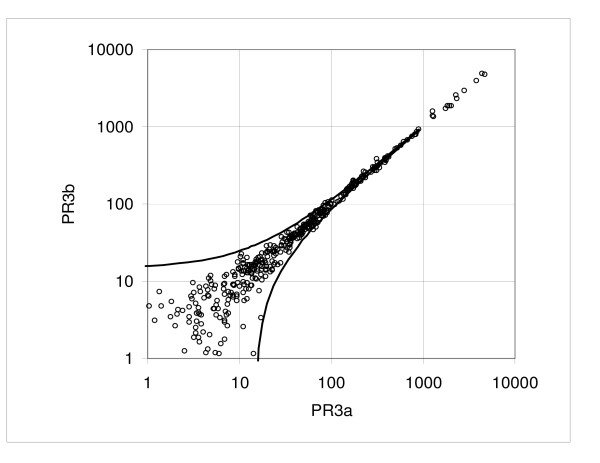
**Example of dispersion in the pooled RNA reference assay: pair PR3a versus PR3b**. Experimental points and boundaries of the 0.9 probability interval.

**Figure 9:**
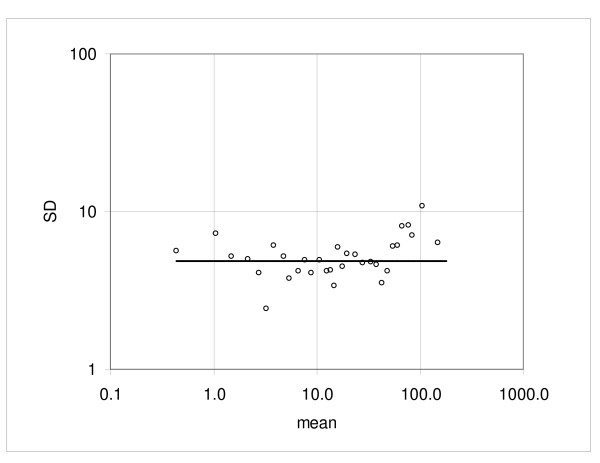
**Example of dispersion in the pooled RNA reference assay: pair PR3a versus PR3b**. Standard deviation calculated from the consecutive samples and the regression curve representing the standard deviation function.

We also looked at the duplicate hybridizations from three independent reverse transcription reactions of a Pooled Reference RNA sample created in our laboratory (pairs PR1a vs. PR1b, PR2a vs. PR2b, PR3a vs. PR3b). The constant term *a *ranged from 2.12 to 4.86 (mean *a *= 3.50) and the proportionality factor *b *ranged from undetectable to 0.082; PR1 and PR2 samples showed quite low variation for both *a *and *b *(typical of other pairs), while the PR3 sample displayed a relatively high constant term *a *= 4.86 with a flat slope value *b *≈ -0.003 (Table 1, Hybridization variation). Figures 6 to 9 show the dispersion plots and standard deviations for the pairs PR1a, b and PR3a, b; the pair PR2 is similar to PR1 (not shown). The plot of the PR3 pair indicates that there is very little change in the SD when the mean intensity increases. It is unknown what might have caused this effect but it may be due to some technical difference in the way these samples were handled.

### Hybridization and transcription variation

Variation in the data sets obtained from the replicate analysis of the three independent aliquots of the Pooled Reference RNA sample should be affected by both hybridization and reverse transcription. Analyzing dispersion for all combinations of PR1a, PR2a, and PR3a (Series PRa), and PR1b, PR2b and PR3b (Series PRb) we observe that mean values for the constant *a *(Series PRa, *a *= 3.55, Series PRb, *a *= 3.73) are similar to the mean values from duplicate comparisons. However, the proportionality coefficients (*b*) are 3–4 fold greater (0.12 and 0.15 versus 0.04) than for duplicate hybridizations (Table 1, Transcription and hybridization variation). Thus, while these values are still low, the reverse transcription reaction clearly introduces meaningful increases in variation.

### Biological replicates

Analysis of the parallel biological cultures comprised all pair-wise combinations of the data from the 1^st ^hybridization (Series Ca, 5 parallel cultures; C1a-C5a) and all pair-wise combinations of the data from the 2^nd ^hybridization (Series Cb). Mean values of the intercept *a *were 3.86 and 3.08, close to the means obtained for the between-duplicate hybridization comparisons. However, the means of the coefficients of proportionality *b *= 0.130 and 0.136 are about three-fold larger than the mean of the between-hybridization coefficients (Table 1, Biological replicates, Series Ca, all pair-wise hyb. and Series Cb, all pair-wise hyb., respectively). Thus, as previously observed for Affymetrix arrays [23], the intercept appears to reflect a measure of technical variability, associated with variability of technological processes (e.g. efficiency of hybridization and labeling, fluctuation of luminescence) or with some features of the array or array reader instrument (e.g. backscatter, scanning and light detection), and exhibits a certain degree of independence of the sample origin. On the other hand, the proportionality coefficient reflects mainly differences in sample origin and composition

The consecutive sampling analysis used throughout this study derives the standard deviation function from the difference of expressions of pairs of ranked genes. Having five replicates for each series gives us an opportunity to verify whether the standard deviation function obtained from the consecutive samples agrees with the function derived from individual genes. Values of the coefficients obtained from the five individual arrays Ca are *a *= 3.39 and *b *= 0.120 (Table 1, Biological replicates, Series Ca, based on individual genes); this is about -12.0% and -7.4% below the consecutive sampling averages, respectively. For the series Cb we get from the individual genes *a *= 3.00 and *b *= 0.116 (Table 1, Series Cb); the differences are -2.6% and -14.8%. The mean differences of the coefficients *a *and *b *for both series are then -7.3% and -11.1%, respectively. Since the probe sets in consecutive samples belong to populations with different, albeit very similar, means, the higher values of the coefficients obtained from the consecutive sampling are to be expected.

### Analysis of the glucose oxidase treated samples

Biological replicates of cell line GM12831 were either untreated (GN1, GN2) or treated with glucose oxidase (GO1, GO2). This low-dose oxidant exposure represents a physiological level of oxidative stress with no apparent induction of toxicity to the cells. After 24 hrs cells were harvested and RNA was extracted. Each of these four samples was hybridized in duplicate to Illumina fiber optic bead arrays (GN1a, GN1b, etc.).

In order to evaluate the quality and consistency of the biological and hybridization replicates, we first performed pair-wise dispersion analysis of the same-type samples (untreated vs. untreated and treated vs. treated). The mean values *a*_*avg *_= 2.7, 2.3 and *b*_*avg *_= 0.059 and 0.062 are very close to the means obtained from duplicate hybridization assays, although in this case the means also include comparisons across biological replicate cultures. For the glucose oxidase treated versus untreated comparisons, we observe that the average constant component *a*_*avg *_= 2.4, is quite similar to values observed for other lymphoblast culture replicates but the proportionality coefficient *b*increases about 2-fold to 0.120 (Table 2; for the complete data see Additional file 7: Supplemental Table S1 and Additional file 8: Supplemental Table S2). To assess how closely the characteristic standard deviation function correlates with the standard deviation values across the range of expressions we determined the correlation coefficient R-square and the standard errors of the coefficients *a *and *b *for seven particular cases: individual genes, series Ca and Cb, pair-wise comparison of the combined data Ca versus Cb and pairs GN1a-GO1a, GN1b-GO1b, GN2a-GO2a and GN2b-GO2b. The mean R-square was 0.78, the mean standard errors of the coefficients *a *and *b *were 4.3% and 8.9%, respectively, and the probability that the coefficients are zero was less than 0.001 (SigmaStat software). Table 2 also shows the *K*_*α *_coefficients that bound the interval containing 90% of values (se the Methods section, Method of analysis). Boundaries of the probability intervals are useful in searching for genes with significantly different expression.

**Table 2:** Dispersion parameters for case/control comparisons. First two data columns show the coefficients of standard deviation function *a *and *b*. The last column labeled Kα shows the coefficient defining boundaries of the probability interval 0.9; CV is the coefficient of variation.

Pair	*a*	*b*	Kα
Untreated (GN1) vs. Untreated (GN2):			
means (n = 4)	2.7	0.059	2.00
CV	0.25	0.32	0.10
Treated (GO1) vs. Treated (GO2):			
means (n = 4)	2.3	0.062	1.84
CV	0.05	0.30	0.05
Untreated (GN1, GN2) vs. Treated (GO1, GO2):			
means (n = 8)	2.4	0.120	2.13
CV	0.19	0.11	0.09

**Figure 10:**
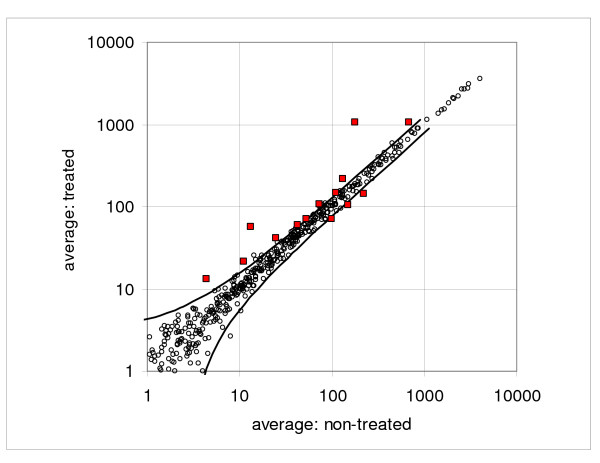
**Dispersion plot of the treated versus non-treated averages in the glucose oxidase assay**. Figure shows the experimental points and boundaries of the 0.95 probability interval (solid lines). Fourteen points selected by the consecutive sampling and coincidence test are shown as enlarged squares.

Differences between the treated and non-treated cells are small. Looking at the plot of the average values, just about three genes are substantially above the random dispersion pattern and none below (Figure 10). However, when we examined all 16 pair-wise comparisons, we found seven genes above the 0.9 probability interval in 14 out of 16 cases and only two below. When we reduced the width of the interval to 0.8, we found 11 genes upregulated and three downregulated; the selected genes are shown in Table 3. While this experiment was not designed to provide a definitive biological demonstration of oxidative stress-induced gene expression, it is encouraging that this analysis has identified six genes known to be altered following oxidative stress (*HMOX1*, *NQO1*, *TFRC*, *P21*, *MGST1*, *CCL5*) and five genes clearly related to repair of DNA damage (*P21*, *GADD45*, *DDB2*, *XPC*, *ATF3*).

**Table 3:** Differentially expressed genes selected by the consecutive sampling and coincidence method. List of the probe sets selected by the consecutive sampling and coincidence method using the interval 0.8. The fold change is calculated using the threshold of 10, substituting into the denominator function max(10, *Y*_*avg*_), where *Y*_*avg *_is the mean of the under-expressed values. Bold print indicates the probe sets selected also for the interval 0.9. The probe set GI_4755127 printed in italics showed an inconsistent behavior in comparison of the probe 1 versus probe 2.

				Untreated		Treated		Fold Change
	Illumina probe set	Gene Name	Gene Function	Mean Intensity	CV	Mean Intensity	CV	Treated/Untr. mean

Upregulated	**GI_4504436**	*HMOX1*	Oxidative Stress	178.9	0.03	1060.1	0.07	5.92
	**GI_4505414**	*NQO1*	Oxidative Stress	13.3	0.09	56.7	0.06	4.27
	**GI_9790904**	*GADD45*	Cell cycle/DNA Repair	11.1	0.26	21.6	0.10	1.95
	**GI_4557514**	*DDB2*	Cell cycle/DNA Repair	24.7	0.07	41.8	0.06	1.69
	**GI_4507456**	*TFRC*	Oxidative Stress/Iron regulation	130.7	0.15	217.1	0.01	1.66
	**GI_17978494**	*P21*	Oxidative Stress/DNA Repair	676.9	0.01	1069.7	0.02	1.58
	**GI_22035635**	*MGST1*	Oxidative Stress	72.4	0.04	107.1	0.09	1.48
	GI_20127459	*XPC*	DNA Repair	42.5	0.07	59.8	0.04	1.41
	GI_4502884	*CLK3*	Cell Cycle	52.3	0.09	71.7	0.03	1.37
	GI_5174726	*TCP1*	Molecular Chaperone	109.5	0.09	147.6	0.05	1.35
	*GI_4755127*	*ATF3*	Cell Cycle/DNA Repair	*4.3*	*0.09*	*13.2*	*0.21*	*1.32*

Downregulated	**GI_4826773**	*G1P2*	Unknown	220.3	0.07	143.1	0.07	1.54
	**GI_22538813**	*CCL5*	Chemokine/Oxidative Stress	147.9	0.02	104.1	0.08	1.42
	GI_4506844	*CCL4*	Chemokine/inflammatory response	98.7	0.04	71.4	0.11	1.38

**Figure 11:**
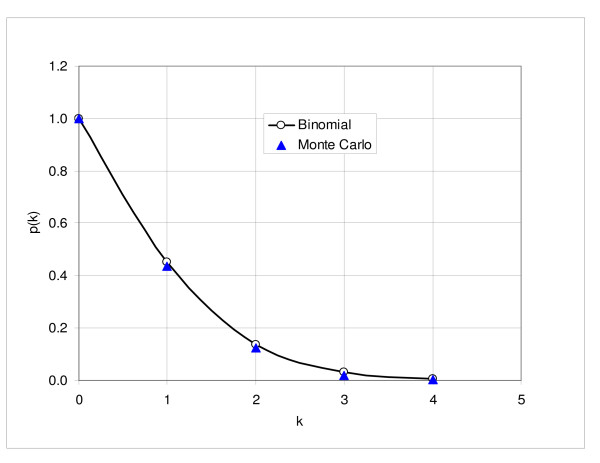
**Probability of observing at least k genes in three trials**. Figure shows the number of probe sets observed in all three trials, assuming that only random differences exist among the gene expressions. Each trial selects randomly 63 probe sets out of 632, corresponding to the number of the probe sets above (below) the 0.8 probability interval. The circles and solid line correspond to the calculations based on binomial probability and triangles represent the results of Monte Carlo simulations (400 runs).

We can estimate probability of false positives from probability of coincidence in three random trials, assuming zero hypothesis, i.e. assuming that the differences are purely random. Figure 11 shows probability of observing *k *or more genes in all three trials for the probability interval 0.8; the circles and solid line correspond to the results obtained using binomial distribution and triangles represent the results of Monte Carlo simulations with four hundred runs. Probability of detecting at least two upregulated or downregulated genes is about 14%; for three or more genes it is 3%.

### Corroboration of the selected differentially expressed genes

We used several independent approaches to assess reproducibility of the results of our data analysis. First we ask the question: Having four replicates, what results we would have obtained, if we only had three pairs of samples? To find the answer we selected the genes above the 0.8 and 0.9 intervals in four sets of three samples and counted the genes common to at least seven out of nine possible combinations. The average percentages of the common genes for any pair of the three-sample tests were 83 and 95, respectively. Surprisingly, the percentage did not decrease, when we reduced the width of the interval (Table 4). In the second verification we submitted the selected genes to the t-test and Wilcoxon test. For the t-test we choose the levels of 0.01 and 0.001, which yielded 65 and 21 over-expressed genes and 4 and 1 under-expressed, respectively. Table 5 shows the comparisons: all 14 genes selected by the coincidence satisfied the Wilcoxon test (P = 0.03) and ten and nine over-expressed genes agree with the t-test at the levels of 0.01 and 0.001, respectively. Third, we checked, how are the selected genes distributed on the plot of average values. We counted 16 and 8 genes above and below the 0.95 interval, respectively: all 14 selected genes are included in these two subsets (Table 5).

**Table 4:** Summary of the test results for 3-sample sets. Results of the reproducibility test. The first and second column list the number of genes identified by the coincidence method for the interval 0.9 and 0.8, respectively, and the third column the number of genes that satisfied the two-tail t-test. The first row shows the mean number of genes that passed the test in complete set (four treated and four untreated samples, 12 coincidences out of 16). The second and third rows, first two columns, give the mean number of genes that passed in seven out of nine comparisons in three-sample sets and the mean of the genes passing concurrently in two particular tests, respectively; the third column shows the mean number of genes that passed the t-test in three-sample sets and the mean of the genes passing concurrently in any two particular tests, respectively, the fourth column corresponds to results obtained for the variance stabilization, fifth for the starred logarithm transformation, sixth for the CyberT method and seventh for the Tusher's calculations. The fourth row shows the ratio of the third row versus the second row in percent.

Probability interval	Coincidence interval 0.9	Coincidence interval 0.8	t-test p = 0.0028	Variance stabilization p = 0.0025	Starred log p = 0.003	CyberT p = 0.0001	Tusher p = 0.023
4-samples test	7	11	27	23	28	17	12
Mean of 3-samples test	12.3	17.5	11.8	11.3	12.3	12.0	12.5
Common to 2 sets (avg)	10.2	16.7	6.3	6.0	6.2	8.7	8.8
Ratio %	83.0	95.2	53.9	53.3	50.3	72.3	70.7

**Table 5:** Results of the Wilcoxon test, t-test and the average plot for the selected differentially expressed genes. Verification of the consecutive sampling and coincidence test by comparison to the Wilcoxon test (P = 0.03), t-test at the level of 0.001 and 0.01 and the and plot of average values. The first five rows of data show the numbers of genes satisfying a given test and the last four the number of genes selected by the coincidence test, which also satisfied a particular second criterion

Probability interval	0.9		0.8	
Upregulated/downregulated	Up	down	up	down

Coincidence: 14 of 16 comparisons	7	2	11	3
Wilcoxon, P = 0.03	65	22	65	22
t-test, P = 0.001	21	1	21	1
t-test, P = 0.01	65	4	65	4
Average plot, no. of genes beyond 0.95 interval	16	8	16	8
Coincident genes that satisfied Wilcoxon	7	2	11	3
Coincident genes that satisfied t-test, P = 0.001	5	0	9	0
Coincident genes that satisfied t-test, P = 0.01	6	0	10	0
Coincident genes beyond 0.95 interval	7	2	11	3

One of the indicators of reliability of the data is consistency of the individual probes within the probe set. Since we have only two probes per a probe set (with one exception), we can only check for consistency of the probe pair behavior. We calculated ratios of the probe set averages for non-treated and treated samples and compared these to the corresponding ratios obtained for individual probes. The difference ranges from -12% to +8%, except for the gene GI_4755127 probe 1, where we got 33% (Table 6). Also, the differences in coefficients of variation of non-treated and treated samples for probes 1 and 2 of the gene GI_4755127 are 0.43 and 0.40, respectively, while the maximum difference for the remaining genes is 0.23. The probe set GI_4755127 was included only among the genes obtained for the 0.8 interval and has the lowest treated-samples average of 13.2.

**Table 6:** Comparison of the signal of first and second probe. Comparison of the probe 1 versus probe 2, nt and tr stand for "non-treated" and "treated," respectively and CV is the coefficient of variation. The ratio pr1/pr1&2 is the fold difference obtained using probe 1 only versus the fold difference obtained from the probe set; similarly for pr2/pr1&2. The last two columns give the absolute values of the difference of coefficient of variation. The minimum and maximum values in the last two rows were calculated excluding the gene GI_4755127, which shows abnormal behavior (printed in italics). Fourteen genes listed were obtained using the interval 0.8; 11 genes printed in bold are the genes, obtained for the interval 0.9*) Excluding the probe set GI_4755127.

Probe Set	Gene Name	Avg(Tr)/Avg(Nt)	Ratio (probe1/probe1&2)	Ratio (probe2/probe1&2)	Probe 1 |CV(nt)-CV(tr)|	Probe 2 |CV(nt)-CV(tr)|
**GI_4504436**	*HMOX1*	5.61	0.99	1.08	0.02	0.01
**GI_4505414**	*NQO1*	4.12	1.05	0.97	0.03	0.12
*GI_4755127*	*GADD45*	*2.78*	*0.67*	*1.11*	*0.43*	*0.40*
**GI_9790904**	*DDB2*	1.84	0.97	1.05	0.03	0.23
**GI_4557514**	*TFRC*	1.62	0.97	1.01	0.19	0.20
**GI_4507456**	*P21*	1.58	1.05	0.96	0.03	0.02
**GI_17978494**	*MGST1*	1.52	1.04	0.98	0.01	0.02
**GI_22035635**	*XPC*	1.41	1.00	1.01	0.03	0.11
GI_20127459	*CLK3*	1.34	1.03	0.92	0.11	0.07
GI_4502884	*TCP1*	1.30	0.99	1.06	0.14	0.01
GI_5174726	*ATF3*	1.27	1.05	1.00	0.08	0.13
GI_4506844	*G1P2*	0.69	1.07	0.95	0.03	0.04
**GI_22538813**	*CCL5*	0.67	1.01	0.96	0.04	0.06
**GI_4826773**	*CCL4*	0.62	1.05	0.88	0.04	0.04

min *		---	0.97	0.88	0.01	0.01
max *		---	1.07	1.08	0.19	0.23

We also compared our candidate genes with the genes selected by the Illumina custom method. Additional file 9 (Supplemental Table A3) shows the list of Illumina genes, including the average gene expression, coefficient of variation and differential score; according to the Illumina scoring, the value of 20 corresponds approximately to P = 0.01. In distinction to all other tests, the Illumina method selects approximately the same number of up- and down-regulated genes: 16 and 15, respectively. All the genes selected by the coincidence test are also found on the Illumina list. There is a good agreement between both methods with the coincidence test, apparently, providing a more rigorous criterion. Of note, the Illumina custom method identified a number of additional genes that are good candidates for regulation by oxidative stress including genes in DNA repair, cell cycle and inflammatory response.

### Comparison of the coincidence test to alternative methods

To assess performance of the coincidence method in the context of other methods currently employed, we compared reproducibility of the coincidence results to the standard t-test, t-test on the variance-stabilized data [32], on the data subjected to "starred logarithm" transformation [38], CyberT method [24] and the method suggested by Tusher and coworkers [30]. We used the same procedure as above, i.e. we identified the probe sets satisfying the probability threshold for four subsets of three microarrays and calculated the average agreement between the all combinations of two trials. For each method we chose significance level that produced a similar number of genes as the coincidence test for the probability interval 0.9.

The mean number of samples satisfying the standard t-test for p < 0.0028 was 11.8, marginally below the average of 12.3, obtained for the coincidence test. At average, only 6.3 were common to any pair of the three-sample tests, representing just about 54% agreement (Table 4). This is to compare with 83% and 95%, attained with the coincidence test. The tests applied to the variance-stabilized data and to the data subjected to the starred logarithm transformation yielded similar performance as the standard t-test, namely 53% at p < 0.0025 and 50% at p < 0.003, respectively. The CyberT method and Tusher's approach showed performance similar to the coincidence test. The average agreement for the former was 8.7 out of 12.0, corresponding to 72% at p < 0.0001; for the latter we obtained 8.8 out of 12.5, corresponding to 71% at p < 0.023 (Table 4).

## Discussion

### Properties of the dispersion patterns

Analysis of variability of the Illumina replicates has shown that experimental frequency distributions are very close to the normal distribution. However, about 5–10% of the samples deviate from normality and include genes with significantly outlying expressions. This implies that any parametric method should be used with caution. At the low end of the expression range the standard deviation is approximately constant, while at the high end it is proportional to the mean expression. The distribution functions of the observed values are symmetrical with respect to the zero axis and the distributions at the right-hand and left-hand sides are equivalent. We demonstrated that the linear standard deviation function provides a good approximation of the overall variability across the array. The intercept is dominant at the low expression level and reliable characterization of the near-zero variability is needed to determine its magnitude. We noted that the values of intercept were similar in all three sets of comparisons, while the coefficient of proportionality in transcription variation and biological replicates was at average about 2 to 3 fold larger as compared to the hybridization variation.

Approximation of the standard deviation across array, provided by the characteristic function derived from the consecutive sampling, was compared to the standard deviation function, derived from the individual genes: the difference for the coefficients *a *and *b *in two independent tests was in the range -3% to -12% and -8% to -14%, respectively. It is understandable that the standard deviation of individual genes is lower, because in the consecutive method we use in each sample elements coming from different populations with small but finite differences in population means. Since the standard deviation increases with the expression mean, this introduces an additional component into the standard deviation estimate.

### Differentially expressed genes in lymphoblasts exposed to glucose oxidase

We created a physiological state of oxidative stress by using a low-dose exposure to glucose oxidase. Previous experiments (data not shown) suggested that this dose could induce oxidative stress genes and produce levels of DNA damage that could be repaired with no apparent cellular toxicity. Indeed, among the differentially expressed genes identified between treated and untreated cells (Table 3), we observe six genes known to be altered following oxidative stress (*HMOX1*, *NQO1*, *TFRC*, *P21*, *MGST1*, *CCL5*) and 5 genes clearly related to repair of DNA damage (*P21*, *GADD45*, *DDB2*, *XPC*, *ATF3*). Additional work is needed to characterize the biological importance of these small changes in gene expression following low-dose oxidative stress.

## Conclusion

In this analysis we examined the frequency distributions of the data in replicate experiments. We demonstrated plausibility of the two-component representation of the standard deviation and showed equivalence of the consecutive sampling method and gene-by-gene evaluation of the standard deviation function. We used the consecutive sampling and coincident test to identify the best candidates among the differentially expressed genes in the samples under oxidative stress; the results were in agreement with the t-test and Wilcoxon statistic and with the Illumina proprietary method. A practical advantage of the consecutive sampling and coincidence approach is that it provides detail information about characteristics of each individual array. Complete pair-wise comparisons can identify the atypical samples and enable the experimenter to decide about their treatment.

The main conclusions can be summarized in the following points:

• Random variability exhibits the Gaussian characteristics; at the low end the frequency distribution of expression values is close to the normal distribution, while at the high end the distribution of relative values is close to normal. The frequency distribution is symmetrical with respect to zero.

• Standard deviation is well approximated by the linear function of the mean gene expression with a constant term.

• Our observations indicate that the change in biological state of the matter is usually reflected in the proportionality coefficient *b*, while the change in technical parameters is frequently correlated with the coefficient *a*.

• Consecutive sampling provides good estimator of the characteristic standard deviation function.

• Consecutive sampling and coincidence test yielded, under given conditions, more consistent results than the t-test applied directly to the normalized data or data submitted to the variance stabilization and starred logarithmic transformation; the performance of the CyberT method and Tusher's method was similar to the coincidence test. The coincidence selection as a nonparametric approach provides more robust selection criterion and can be used for assays with only duplicate arrays.

## Methods

### Cell culture conditions

Epstein Barr Virus (EBV)- transformed lymphoblastoid cell lines (LCLs) were grown in RPMI-1640 medium, supplemented with 10% fetal bovine serum (Invitrogen, Carlsbad, CA) and 2 mM L-glutamine (Life Technologies, Gaithersburg, MD) at 37°C in a humidified 5% CO_2 _atmosphere. Before treatment with glucose oxidase, cells were diluted to a concentration of 2 × 10^5 ^cells/ml in fresh RPMI-1640 media (plus 15% FBS) and allowed to grow out for 18 hours to condition the media. After 18 hours, 10 ml of suspended cells were aliquotted into Petri dishes. Glucose oxidase (Molecular Probes) was added to test samples at a final concentration of 1 mUnit/ml, while dilution buffer (1 mM sodium acetate) was added to controls. Test and control samples were incubated at standard conditions for 8 or 24 hours. Parallel cell culture samples (biological replicates) were extracted and reverse transcribed separately. RNA was extracted from test and control samples using RNeasy Midi extraction columns, according to the manufacturer's instructions (Qiagen). The pooled reference used in these experiments was a combination of equal amounts of RNA from six LCLs (GM10469, GM10967, GM11321, GM12909, GM13838, and GM14682, Coriell Cell Repositories, Camden, NJ) and from 3 lymphoid tumor lines (L428 (DSMZ, Braunschweig, Germany), and Jurkat and Raji (ATCC, Manassas, VA)).

### Illumina bead-based arrays

The Illumina Gene Expression system was used for direct hybridization of labeled cRNAs to gene-specific 50-mer oligonucleotide probes attached to microbeads. For each sample, 200 ng of total RNA was aliquoted into 1 well of a 96-well plate. Labeled cRNA was produced by a reverse transcription followed by in-vitro transcription according to the manufacturer's instructions (MessageAmp II, Ambion). Duplicate aliquots of each cRNA sample (1 μg cRNA each) were distributed into parallel microwells in a 384 well hybridization plate with buffer, paired with a Sentrix array matrix (SAM), and incubated at 55°C overnight as per the Illumina standard protocol. The following day the SAM was washed, blocked with casein (Pierce, Rockford, IL), and signal was developed with streptavidin-Cy3 using Fluorolink Cy3 (Amersham, Piscataway, NJ) according to the manufacturer's instructions. The SAM was then imaged with the Illumina BeadArray Reader GX.

### Method of analysis

The consecutive sampling method [23] provides a convenient tool for the global characterization of dispersion patterns in pair-wise comparisons. Briefly, the probe sets of a given pair of arrays are ranked according to the mean expression, statistical samples are defined as *k *consecutive pairs (typically *k *= 12) and the standard deviations are calculated from the difference of expressions. The estimator of the characteristic standard deviation function *SD *is then determined by fitting the linear function

*SD *= *a *+ *bY*_*mean*_

to the experimental points at the logarithmic scale; (Eq. (1), *Y*_*m *_is the sample mean). In order to obtain representative standard deviation at a given expression level, the differences in mean expressions of the genes in a given sample must be small. This poses no problem at the low expression end, but at the high expressions the density of genes within a narrow expression interval is small and a certain number of genes (probe sets) must be excluded.

The consecutive sampling program is written in Basic and uses the Excel platform. After sorting and definition of the samples it calculates the standard deviations and determines the characteristic function using the logarithmic transform and nonlinear regression subprogram. Once it determines the standard deviation function, it calculates the boundaries of chosen probability intervals. The upper and lower limits in the dispersion plot *Y*_*2 *_versus *Y*_*1 *_are defined as



and



where *K*_*α *_is a constant corresponding to the probability interval α.

Several "reliability checks" have been incorporated into the consecutive sampling program. First, assuming independent samples we verify the identity

*SD*(*Y*_*diff*_) = *SD*(*Y*_1_) + *SD*(*Y*_2_),     (4)

where *SD*(*Y*_*diff*_) and *SD*(*Y*_*i*_) are the standard deviations calculated from the expression difference of the ordered sample and from the expression values of the array *i*, respectively (see Supplementary Material in Ref. [23]). It provides good verification of variability of the mean within a given sample: here we obtained agreement within about 2%. The second check calculates the average number of samples failing the Kolmogorov-Smirnov normality test (P = 0.05) and the third compares the number of genes beyond the 0.95 probability interval to the number of genes outside the interval corresponding to 1.96 standard deviations (0.95 probability interval of the normal distribution with the same mean and standard deviation). Additional subprograms calculate skewness and kurtosis and assess the symmetry. The program provides the output tables including the verifications, parameters characterizing the dispersion and list of the genes outside specified probability intervals.

## Competing interests

The author(s) declare that they have no competing interests.

## Authors' contributions

Experimental samples were prepared and processed by MCM III and DAB in the Environmental Genomics Section, National Institute of Environmental Health Sciences. Data analysis was carried out by JPN at McGill University and Genome Quebec Innovation Centre.

## Reviewers' comments

### Reviewer's report 1

*Alexander Karpikov, MB&B Department, Yale University, New Haven, CT 06520 USA *(*nominated by MarkGerstein, MB&B Department, Yale University, New Haven, CT 06520 USA*).

Reviewer comments:

I read your article and found it quite interesting. I think it has enough material for the publication. My major comment is on the style. I think the article is very difficult to read and its style should be improved prior to the publication.

**Author response**: *In response to the reviewer's concern we abbreviated the text and introduced number of revisions. First, in the Background discussion we stated the purpose of our study and the main points of the individual sections more clearly. Second, we partially reorganized the text and introduced two new subheadings. We tried to be clearer and more specific and to eliminate deviations from the main subject. Throughout the text we systematically tried to indicate the goal of a given particular section and clearly describe the approach used to achieve it*.

### Reviewer's report 2

Eugene V. Koonin, National Center for Biotechnology Information, National Library of Medicine, National Institutes of Health, Bethesda, MD, USA

Reviewer comments:

I think this is a useful, carefully performed study on microarray analysis statistics. Although the technology behind the experiments analyzed in this paper is not the most common one, the tests investigated here may have general applicability.

### Reviewer's report 3

*King Jordan, National Center for Biotechnology Information, National Institutes of Health, Bethesda, MD 20894*.

Reviewer comments:

In this work, Novak *et al*. analyzed the statistical properties of gene expression level data generated from the Illumina GEX Sentrix™ microarray platform, which employs a fiberoptic bead-based approach to measuring expression levels. Microarray technology is evolving rapidly and the trend is towards increasingly high density arrays that are capable, in principle, of generating expression profiles for multiple replicates of entire eukaryotic genomes from single chips. The bead based technology employed by Illumina arrays represents an experimental methodology that is qualitatively distinct from that employed by the industry leader Affymetrix. In addition to allowing for increased density and multiple samples per chip, the Illumina bead-based arrays afford several other potential advantages including: i-longer 50 mer probes that presumably increase both selectivity and specificity, ii-low sample and reagent volumes that, along with a high density of features on the array, lead to a relatively low price per sample and a consequently more ambitious experimental scale, and iii-high redundancy, with ~30 beads for each oligo, that can be exploited to increase confidence in specific gene expression levels.

While the move to increasingly high density arrays represents a potential boon to researchers, it also presents fundamental bioinformatics challenges regarding the analysis, visualization and interpretation of expression data. Unfortunately, the bioinformatics technology needed to meaningfully comprehend the results of increasingly high throughput gene expression profiling tends to lag behind the new experimental approaches. It is precisely this challenge that Novak *et al*. took up in their analysis of the dispersion patterns of expression data generated by Illumina arrays. To evaluate dispersion patterns, the authors implemented the previously developed consecutive sampling method whereby probe sets are ranked according to mean expression and sets with similar means are binned prior to comparison. They compared dispersion patterns from three groups of samples, each of which allows for different sources of variation – hybridization, reverse transcription and biological – to be considered independently. Evaluation of the assumption of normality revealed distinct deviations at the low and high ranges of expression. Based on this pattern, they found that the standard deviation of the variability can be broken down into two components – a constant term and a proportional term. The constant term dominates at low levels of expression, while the proportional term dominates at high levels. Having established the statistical properties of the expression level dispersion, the authors performed a comparison of gene expression from glucose oxidase treated versus un-treated cell lines in order to identify differentially expressed genes that may play a role in the response to oxidative stress. They were able to identify 11 such genes using the consecutive sampling approach, affirming its potential utility.

By way of critique, one may quibble with the statistical techniques employed by the authors, and there are clearly other tacks that could be taken to analyze this kind of data. For instance, the authors compared their consecutive sampling method to standard parametric (t-test) and non-parametric (Wilcoxon) methods as well as to Illumina's own proprietary method. They show that their consecutive sampling method performs comparably to these methods, in terms of identifying similar sets of differentially expressed genes, and also shows more consistency. It would have been nice to see a more systematic comparison of different methods for selecting differentially expressed genes since this is the essence of what investigators usually want to glean from microarray studies. For example there are a number of methods cited in this paper including several non-parametric approaches, a variance-stabilizing transformation and Bayesian approaches that could have been compared with the authors' method of choice. However, the analysis that is presented in the paper is detailed and thorough.

The impact of the work can also be considered to be somewhat mitigated by the fact that the consecutive sampling method introduced is an extension of the authors own previous work on Affymetrix arrays. The scope of this study was also quite small, expression of only 632 genes was analyzed, and one may wonder whether the dispersion properties for a set of that size would hold up for whole-genome data sets. In addition, while the authors do make some attempt to study an actual biological system – exposure of a cell line to low dose oxidative stress – there is relatively little biological insight that can be gleaned from this work. To be fair however, both substantial utility and novelty can be found in the manuscript, pursuant to the fact that it represents the first study of data variability in the Illumina bead-based microarrays. Thus, the authors have made an important, if tentative, contribution towards bridging the gap between emerging microarray experimental technologies and the bioinformatics tools needed to interpret their output.

**Author response**: *First, we would like to thank Dr. Jordan for very thorough and helpful review. In response to his comment we extended comparisons of reproducibility to include the variance stabilization, "starred logarithm" transformation, CyberT method and Tusher's approach. Regarding the comment that the consecutive sampling method was already introduced in the 2002 publication *[23]: *We would like to mention, that the original paper described only the principle and justification of the approach. On the basis of this principle we developed the method of analysis, incorporating the subroutines calculating the characteristic standard deviation function, boundaries of the probability intervals for selected set of values, tests of normality, calculations of skewness and kurtosis, etc. (c.f. Methods section). Furthermore, in the present study we introduced the application of the consecutive sampling and probability intervals to the differential expression analysis via coincidence test and presented the estimate of the number of coincidences based on probability of coincidences in random trials. We hope that new information is sufficiently noteworthy to make it interesting to readers. Regarding the experimental part of the study, unfortunately, lack of support makes it impossible now to extend the study to include larger arrays or to make a more thorough investigation of the biological properties of the system under consideration*.

## Supplementary Material

Additional file 1 **Supplemental Figure S1, comparison of the pooled reference samples C5a and C5b before renormalization**. Dispersion pattern and 0.9 probability interval, before normalization. Click here for file

Additional file 2 **Supplemental Figure S2, comparison of the pooled reference samples C5a and C5b before renormalization**. Running mean of 90 genes, before renormalization. Click here for file

Additional file 3 **Supplemental Figure S3, comparison of the pooled reference samples C5a and C5b after renormalization**. Dispersion pattern and 0.9 probability interval, after normalization. Click here for file

Additional file 4 **Supplemental Figure S4, comparison of the pooled reference samples C5a and C5b after renormalization**. Running mean of 90 genes, after renormalization. Click here for file

Additional file 5 **Supplemental Figure S5, quantile-quantile plot of the frequency distribution**. Comparison of the observed expressions with the corresponding inverse normal distribution, combined samples C1a, C2a, C3a, C4a, C5a: range of average expressions from 0.1 to 2.0; figure shows the relative values (expressions divided by the mean of five arrays). Click here for file

Additional file 6 **Supplemental Figure S6, quantile-quantile plot of the frequency distribution**. Comparison of the observed expressions with the corresponding inverse normal distribution, combined samples C1a, C2a, C3a, C4a, C5a: range of average expressions from 117 to 5432. Click here for file

Additional file 7 **Supplemental Table S1, same-type sample dispersion parameters for the glucose oxidase treatment assay**. Coefficients of the standard deviation function *a *and *b *and *K*_*α *_coefficient corresponding to 0.9 probability interval; nt and tr stand for "untreated" and "treated," respectively. Click here for file

Additional file 8 **Supplemental Table S2, dispersion parameters for case/control comparisons in the glucose oxidase treatment assay**. Coefficients of the standard deviation function *a *and *b *and *K*_*α *_coefficient corresponding to 0.9 probability interval; nt and tr stand for "un-treated" and "treated," respectively. Click here for file

Additional file 9 **Supplemental Table S3, differentially expressed genes selected by the Illumina custom algorithm**. Probe sets selected by the Illumina method. The table shows the gene name and function, mean intensity, coefficient of variation and Illumina differential score; value ± 20 corresponds to P = 0.01. Note that Illumina uses different normalization and, consequently, the mean intensities of Tables 3 and 4 do not agree. Bold print indicates the probe sets selected by the consecutive sampling method and coincidence test. Click here for file
