# Supplementary material for: Variation in fiberoptic bead-based oligonucleotide microarrays: dispersion characteristics among hybridization and biological replicate samples
Source: Biol Direct. 2006 Jun 20;1:18. doi: 10.1186/1745-6150-1-18 (PMC1533816; doi:10.1186/1745-6150-1-18)
Supplement: Additional file 1 — Supplemental Figure S1, comparison of the pooled reference samples C5a and C5b before renormalization. Dispersion pattern and 0.9 probability interval, before normalization. [file 1745-6150-1-18-S1.doc]

## Additional file 1 – Supplemental Figure S1 -- Comparison of the pooled reference samples C5a and C5b before renormalization

## Dispersion pattern and 0.9 probability interval, before normalization.
