# Supplementary material for: Variation in fiberoptic bead-based oligonucleotide microarrays: dispersion characteristics among hybridization and biological replicate samples
Source: Biol Direct. 2006 Jun 20;1:18. doi: 10.1186/1745-6150-1-18 (PMC1533816; doi:10.1186/1745-6150-1-18)
Supplement: Additional file 4 — Supplemental Figure S4, comparison of the pooled reference samples C5a and C5b after renormalization. Running mean of 90 genes, after renormalization. [file 1745-6150-1-18-S4.doc]

## Additional file 4 – Supplemental Figure S4, comparison of the pooled reference samples C5a and C5b after renormalization

## Running mean of 90 genes, after renormalization.
