# Supplementary material for: Variation in fiberoptic bead-based oligonucleotide microarrays: dispersion characteristics among hybridization and biological replicate samples
Source: Biol Direct. 2006 Jun 20;1:18. doi: 10.1186/1745-6150-1-18 (PMC1533816; doi:10.1186/1745-6150-1-18)
Supplement: Additional file 5 — Supplemental Figure S5, quantile-quantile plot of the frequency distribution. Comparison of the observed expressions with the corresponding inverse normal distribution, combined samples C1a, C2a, C3a, C4a, C5a: range of average expressions from 0.1 to 2.0; figure shows the relative values (expressions divided by the mean of five arrays). [file 1745-6150-1-18-S5.doc]

## Additional file 5 – Supplemental Figure S5, quantile-quantile plot of the frequency distribution

## Comparison of the observed expressions with the corresponding inverse normal distribution, combined samples C1a, C2a, C3a, C4a, C5a: range of average expressions from 0.1 to 2.0; figure shows the relative values (expressions divided by the mean of five arrays).
