# Supplementary material for: Variation in fiberoptic bead-based oligonucleotide microarrays: dispersion characteristics among hybridization and biological replicate samples
Source: Biol Direct. 2006 Jun 20;1:18. doi: 10.1186/1745-6150-1-18 (PMC1533816; doi:10.1186/1745-6150-1-18)
Supplement: Additional file 6 — Supplemental Figure S6, quantile-quantile plot of the frequency distribution. Comparison of the observed expressions with the corresponding inverse normal distribution, combined samples C1a, C2a, C3a, C4a, C5a: range of average expressions from 117 to 5432. [file 1745-6150-1-18-S6.doc]

## Additional file 6 – Supplemental Figure 6 - Quantile-quantile plot of the frequency distribution

## Comparison of the observed expressions with the corresponding inverse normal distribution, combined samples C1a, C2a, C3a, C4a, C5a6: range of average expressions from 117 to 5432.
