# Supplementary material for: Variation in fiberoptic bead-based oligonucleotide microarrays: dispersion characteristics among hybridization and biological replicate samples
Source: Biol Direct. 2006 Jun 20;1:18. doi: 10.1186/1745-6150-1-18 (PMC1533816; doi:10.1186/1745-6150-1-18)
Supplement: Additional file 7 — Supplemental Table S1, same-type sample dispersion parameters for the glucose oxidase treatment assay. Coefficients of the standard deviation function a and b and Kα coefficient corresponding to 0.9 probability interval; nt and tr stand for "untreated" and "treated," respectively. [file 1745-6150-1-18-S7.doc]

**Additional file 7 – Supplemental Table 7, same-type sample dispersion parameters for the glucose oxidase treatment assay**

Coefficients of the standard deviation function *a* and *b* and *K*_α_ coefficient corresponding to 0.9 probability interval; nt and tr stand for “untreated” and “treated,” respectively.

| Pair (Nt vs. Nt) | a | b | Kp(0.9) |
| --- | --- | --- | --- |
| GN1a vs GN1b | 3.7 | 0.035 | 1.88 |
| GN1a vs GN2a | 3.0 | 0.049 | 2.09 |
| GN1a vs GN2b | 2.4 | 0.081 | 1.82 |
| GN2a vs GN1b | 3.0 | 0.057 | 2.17 |
| GN2a vs GN2b | 1.8 | 0.050 | 2.27 |
| GN1b vs GN2b | 2.2 | 0.083 | 1.78 |
| average | 2.7 | 0.059 | 2.00 |
| CV | 0.25 | 0.32 | 0.10 |
| Pair (Tr vs. Tr) | a1 | a2 | Kp(0.9) |
| GO1a vs GO1b | 2.2 | 0.050 | 1.75 |
| GO1a vs GO2a | 2.5 | 0.057 | 1.85 |
| GO1a vsGO2b | 2.4 | 0.085 | 1.85 |
| GO2a vs GO1b | 2.4 | 0.072 | 1.74 |
| GO2a vs GO2b | 2.4 | 0.034 | 1.97 |
| GO1b vs GO2b | 2.2 | 0.073 | 1.87 |
| average | 2.3 | 0.062 | 1.84 |
| CV | 0.05 | 0.30 | 0.05 |
