# Supplementary material for: Variation in fiberoptic bead-based oligonucleotide microarrays: dispersion characteristics among hybridization and biological replicate samples
Source: Biol Direct. 2006 Jun 20;1:18. doi: 10.1186/1745-6150-1-18 (PMC1533816; doi:10.1186/1745-6150-1-18)
Supplement: Additional file 8 — Supplemental Table S2, dispersion parameters for case/control comparisons in the glucose oxidase treatment assay. Coefficients of the standard deviation function a and b and Kα coefficient corresponding to 0.9 probability interval; nt and tr stand for "un-treated" and "treated," respectively. [file 1745-6150-1-18-S8.doc]

**Additional file 8** – **Supplemental Table S2, dispersion parameters for case/control comparisons in the glucose oxidase treatment assay**

Coefficients of the standard deviation function *a* and *b* and *K*_α_ coefficient corresponding to 0.9 probability interval; Nt and Tr stand for “un-treated” and “treated,” respectively.

| Pair (Nt vs. Tr) | a | b | Kα |
| --- | --- | --- | --- |
| GN1A-GN2A | 3.1 | 0.116 | 2.18 |
| GN1A-GO1B | 3.0 | 0.118 | 2.24 |
| GN1A-GO2A | 3.2 | 0.117 | 2.16 |
| GN1A-GO2B | 3.0 | 0.141 | 2.14 |
| GN1B-GN2A | 2.7 | 0.119 | 2.29 |
| GN1B-GO1B | 2.4 | 0.116 | 2.40 |
| GN1B-GO2A | 2.5 | 0.121 | 2.25 |
| GN1B-GO2B | 2.6 | 0.122 | 2.25 |
| GO1A-GN2A | 2.3 | 0.104 | 2.32 |
| GO1A-GO1B | 2.2 | 0.119 | 2.26 |
| GO1A-GO2A | 2.4 | 0.132 | 2.20 |
| GO1A-GO2B | 2.3 | 0.152 | 2.04 |
| GN2B-GN2A | 1.9 | 0.098 | 1.91 |
| GN2B-GO1B | 1.7 | 0.102 | 1.90 |
| GN2B-GO2A | 1.9 | 0.118 | 1.78 |
| GN2B-GO2B | 1.7 | 0.128 | 1.77 |
| average | 2.4 | 0.120 | 2.13 |
| CV | 0.19 | 0.11 | 0.09 |
