# Supplementary material for: Variation in fiberoptic bead-based oligonucleotide microarrays: dispersion characteristics among hybridization and biological replicate samples
Source: Biol Direct. 2006 Jun 20;1:18. doi: 10.1186/1745-6150-1-18 (PMC1533816; doi:10.1186/1745-6150-1-18)
Supplement: Additional file 9 — Supplemental Table S3, differentially expressed genes selected by the Illumina custom algorithm. Probe sets selected by the Illumina method. The table shows the gene name and function, mean intensity, coefficient of variation and Illumina differential score; value ± 20 corresponds to P = 0.01. Note that Illumina uses different normalization and, consequently, the mean intensities of Tables 3 and 4 do not agree. Bold print indicates the probe sets selected by the consecutive sampling method and coincidence test. [file 1745-6150-1-18-S9.doc]

## Additional file 9 – Supplemental Table S3, differentially expressed genes selected by the Illumina custom algorithm.

Probe sets selected by the Illumina method. The table shows the gene name and function, mean intensity, coefficient of variation and Illumina differential score; value ±20 corresponds to P = 0.01. Note that Illumina uses different normalization and, consequently, the mean intensities of Tables 3 and 4 do not agree. Bold print indicates the probe sets selected by the consecutive sampling method and coincidence test.

|  |  |  |  | Untreated | | Treated | | Significance |
| --- | --- | --- | --- | --- | --- | --- | --- | --- |
|  | Illumina probe set | Gene Name | Gene Function/description | Mean Intensity | CV | Mean Intensity | CV | Differential score |
| Upregulated | **GI_4504436** | ***HMOX1*** | **Oxidative Stress** | **349.2** | **0.03** | **1944.1** | **0.04** | **371.3** |
|  | **GI_4505414** | ***NQO1*** | **Oxidative Stress** | **24.5** | **0.10** | **101.0** | **0.06** | **286.7** |
|  | **GI_17978494** | ***P21*** | **Oxidative Stress/DNA Repair** | **1295.0** | **0.02** | **1962.4** | **0.03** | **144.2** |
|  | **GI_4507456** | ***TFRC*** | **Oxidative Stress/Iron regulation** | **252.1** | **0.12** | **406.3** | **0.02** | **128.3** |
|  | **GI_22035635** | ***MGST1*** | **Oxidative Stress** | **139.5** | **0.04** | **197.8** | **0.10** | **50.4** |
|  | **GI_4557514** | ***DDB2*** | **Cell cycle/DNA Repair** | **47.0** | **0.06** | **75.1** | **0.06** | **49.8** |
|  | **GI_4755127** | ***ATF3*** | **Cell Cycle/DNA Repair** | **7.6** | **0.21** | **24.2** | **0.24** | **34.9** |
|  | **GI_20127459** | ***XPC*** | **DNA Repair** | **81.7** | **0.07** | **108.3** | **0.05** | **31.4** |
|  | **GI_4502884** | ***CLK3*** | **Cell Cycle** | **100.0** | **0.09** | **131.4** | **0.05** | **31.0** |
|  | GI_5803180 | *STIP1* | stress-induced-phosphoprotein 1 | 144.6 | 0.06 | 181.5 | 0.04 | 30.9 |
|  | GI_6912519 | *MUTYH* | mutY homolog | 229.7 | 0.03 | 277.9 | 0.03 | 27.1 |
|  | GI_21614519 | *G6PD* | glucose-6-phosphate dehydrogenase | 153.2 | 0.05 | 191.5 | 0.09 | 26.8 |
|  | **GI_5174726** | ***TCP1*** | **Molecular Chaparone** | **212.3** | **0.11** | **275.7** | **0.04** | **26.2** |
|  | **GI_9790904** | ***GADD45*** | **Cell cycle/DNA Repair** | **20.0** | **0.35** | **38.4** | **0.13** | **26.1** |
|  | GI_16357478 | *CDC37* | CDC37 cell division cycle 37 homolog | 433.5 | 0.07 | 473.1 | 0.05 | 23.2 |
|  | GI_16357491 | *CDC2L2* | cell division cycle 2-like 2 | 69.0 | 0.07 | 89.0 | 0.06 | 20.6 |
| Down Reg | **GI_4826773** | ***G1P2*** | **Unknown** | **428.1** | **0.06** | **265.2** | **0.06** | **-117.6** |
|  | **GI_22538813** | ***CCL5*** | **Chemokine/Oxidative Stress** | **287.5** | **0.04** | **191.3** | **0.09** | **-88.0** |
|  | **GI_4506844** | ***CCL4*** | **Chemokine/** | **189.8** | **0.04** | **129.2** | **0.12** | **-58.2** |
|  | GI_10835060 | *ID3* | inflammatory response | 218.4 | 0.03 | 163.0 | 0.02 | -55.4 |
|  | GI_4505812 | *DNCL1* | dynein, cytoplasmic, light polypeptide 1 | 885.7 | 0.06 | 701.1 | 0.06 | -47.0 |
|  | GI_13259540 | *UCP2* | uncoupling protein 2 | 534.1 | 0.02 | 407.9 | 0.06 | -41.3 |
|  | GI_19923751 | *RPA3* | replication protein A3, 14kDa | 464.4 | 0.08 | 368.1 | 0.05 | -39.6 |
|  | GI_13325059 | *CYP1B1* | cytochrome P450, family 1, subfamily B | 242.7 | 0.04 | 177.4 | 0.11 | -33.8 |
|  | GI_18641376 | *HLA-DMB* | major histocompatibility complex, class II | 1038.1 | 0.08 | 825.2 | 0.03 | -27.9 |
|  | GI_330368 | *HS4IETGA* | Epstein-Barr virus mRNA encoding with BZLF1 | 162.7 | 0.04 | 127.5 | 0.06 | -27.4 |
|  | GI_4505232 | *MPG* | N-methylpurine-DNA glycosylase | 58.0 | 0.04 | 41.1 | 0.11 | -27.0 |
|  | GI_20127449 | *PRKCB1* | protein kinase C, beta 1 | 163.8 | 0.05 | 131.3 | 0.02 | -24.4 |
|  | GI_14327895 | *CCNB1* | cyclin B1 | 268.5 | 0.06 | 228.4 | 0.06 | -22.5 |
|  | GI_10092618 | *NFKBIA* | nuclear factor of kappa light polypeptide gene enhancer in B-cells inhibitor, alpha | 873.2 | 0.05 | 762.0 | 0.09 | -22.0 |
|  | GI_4758253 | *EIF1AY* | eukaryotic translation initiation factor 1A | 110.9 | 0.11 | 86.1 | 0.10 | -21.3 |
